# Tobacco Use and Environmental Smoke Exposure among Taiwanese Pregnant Smokers and Recent Quitters: Risk Perception, Attitude, and Avoidance Behavior

**DOI:** 10.3390/ijerph10094104

**Published:** 2013-09-03

**Authors:** Ming-Cheng Lai, Feng-Sha Chou, Yann-Jy Yang, Chih-Chien Wang, Ming-Chang Lee

**Affiliations:** 1 Graduate Institute of Business Administration, National Taipei College of Business, 321 Jinan Rd., Section 1, Taipei 100, Taiwan; E-Mail: laimc@mail.ntcb.edu.tw; 2 Department of Business Administration, National Taipei University, 151, University Rd., San Shia District, New Taipei City 23741, Taiwan; E-Mail: choufengsha@gmail.com; 3 Department of Business Innovation and Development, MingDao University, 369 Wen-Hua Rd., Pettow, ChangHua 52345, Taiwan; E-Mail: yjyang07@gmail.com; 4 Graduate Institute of Information Management, National Taipei University, 151, University Rd., San Shia District, New Taipei City 23741, Taiwan; 5 Department of Management, Fo Guang University, 160, Linwei Rd., Jiaosi, Yilan County 26247, Taiwan; E-Mail: lmj0055@gmail.com

**Keywords:** tobacco smoking, risk behavior, pregnancy, environmental tobacco smoke pollution

## Abstract

In this study, we conducted an empirical survey of the avoidance behaviors and risk perceptions of active and passive smoking pregnant smokers and recent quitters. We employed an online questionnaire survey by recruiting 166 voluntary participants from an online parenting community in Taiwan. The results of the empirical survey revealed that three-fourths of smokers quit smoking during pregnancy and one-fourth continued smoking. All pregnant women who continued smoking had partners or lived with relatives who smoked. Current smokers and quitters differed significantly in their risk perceptions and attitudes toward smoking during pregnancy. Most pregnant smokers and quitters adopted passive smoking avoidance behaviors at home and in public. Nevertheless, one-fifth of pregnant women chose not to avoid passive smoking. We concluded that most women stop smoking during pregnancy; however, most women continue to be exposed to passive-smoking environments. Perceived fetal health risks and attitudes toward smoking during pregnancy are critical predictors of the anti-smoking behaviors of pregnant women.

## 1. Introduction

Tobacco use and environmental smoke exposure are critical health issues for pregnant women and their unborn babies [1]. Pregnant smokers place themselves and their babies in a high-risk situation [2]. Previous studies have reported that smoking during pregnancy is significantly associated with increased risks of intrauterine growth retardation [3], low birth weight [4], miscarriage [5], stillbirth [6], congenital malformation [6], early weaning [7], sudden infant death syndrome [8], and genetic-related hereditary diseases [9]. Smoking during pregnancy is a complex and variable behavior [10]. “To quit or not to quit” is a crucial but difficult decision for pregnant smokers. Pregnant smokers are typically asked to quit smoking for the health of their babies and themselves; however, not all women choose to quit during pregnancy [11].

To reduce smoking during pregnancy, we must understand the factors influencing the attitudes of pregnant smokers toward smoking. Although numerous researchers have conducted empirical studies reporting the negative effects of smoking during pregnancy [2], pregnant smokers are not necessarily convinced that the risks of smoking threaten their unborn children. By interviewing women who smoked during pregnancy, Haslam and Draper determined that half of pregnant smokers were unaware of the health risks of smoking [12]. Ortendahl and Näsman revealed that pregnant smokers perceived a lesser probability of smoking-related health consequences compared with those who intended to quit [2]. Because they remained unaware of the consequences that smoking could have on fetal health, pregnant smokers did not worry about the health risks of smoking.

Tobacco smoke pollution is induced by both active and passive smoking [13]. Previous studies have revealed that pregnant smokers typically have partners who smoke [11,12]. Penn and Owen indicated that whether a pregnant woman had a partner who smoked was a significant predictor of her current smoking status [11]. Based on the results of interviews, Haslam and Draper reported that almost all pregnant smokers had a partner who smoked [12]. Thus, certain pregnant smokers may choose to continue smoking because their partners or family members smoke. The health of these pregnant women and their fetuses is threatened by both the active smoking and the passive smoking of their partners or families.

Risk perception affects the attempts of pregnant smokers to quit smoking. However, scant literature has addressed the perceptions of pregnant smokers regarding the health risks of smoking and the avoidance of passive smoking. Active and passive smoking during pregnancy is a severe health problem in Taiwan. Previous surveys have indicated that female smokers in Taiwan had low smoking cessation rates during pregnancy and their partners continued using tobacco throughout the pregnancy [14]. Previous studies have also indicated that numerous pregnant Taiwanese women are exposed to environmental tobacco smoke [15,16]. According to the Taiwan Tobacco Control Annual Report 2012, 19.2% of adult Taiwanese females between 20 and 35 years old are smokers [17]. According to the Adult Smoking Behavior Surveillance System Survey conducted by the Taiwan Bureau of Health Promotion of the Department of Health, 17.3% females live in households in which others smoke in their presence, and 6.2% of females are frequently exposed to secondhand smoking in public [18].

Thus, we aim to explore the risk perceptions of both pregnant smokers and quitters in Taiwan regarding smoke, smoking behaviors, and avoidance behaviors toward passive smoking. We sought to answer the following questions: Do pregnant Taiwanese smokers perceive that smoking is associated with fewer fetal health risks compared with quitters? Are pregnant Taiwanese smokers more often exposed to passive smoke at home or in public compared with quitters?

## 2. Method

### 2.1. Participants

The target populations of this study were pregnant smokers and quitters in Taiwan. We collected data by using a convenience sample of pregnant women in an online baby-parenting community. Because more than 95% of Taiwanese women ages 21–40 are Internet users according to a survey by Taiwan Network Information Center [18], the Internet is an appropriate platform for accessing pregnant women in Taiwan.

An online questionnaire was employed in this study during a 2-week period from 25 April 2013 to 7 May 2013. The expected sample size was 700, assuming that the expected percentage of pregnant women who smoked was 20%, given a sampling error of 3% and a 95% confidence interval. All the participants joined the study voluntarily and were informed of their right to decline. The questionnaire required approximately 5 min to completing.

We contacted 712 pregnant women. However, 546 did not smoke; thus, they did not meet the inclusion criteria. Pregnant women who were smokers or quitters were included in the study; pregnant women who had no previous smoking experience or did not report their smoking history were excluded from the study. The remaining 166 pregnant women who smoked before or during pregnancy were included in the data analysis. All participants lived in Taiwan.

### 2.2. Procedure

We employed an online questionnaire by posting a call for voluntary participants in a well-known online community for baby parenting in Taiwan. The participants who completed the online questionnaire received by post mail a small reward (baby clothing and socks) worth approximately $3 US.

### 2.3. Research Ethics

Before participating in the questionnaire survey, the participants signed an online informed consent form indicating that they were aware of the nature of the study, including its benefits and risks. The Research Ethics Committee of National Taiwan University approved the study (approval No. 201301HS011).

### 2.4. Measures

A questionnaire was developed after we reviewed the existing literature on smoking during pregnancy. The questionnaire captured demographic characteristics, gravidity, perceived health risk, attitude toward smoking during pregnancy, smoking history, smoking volume (before and during pregnancy), exposure to passive smoking, and avoidance behaviors toward passive smoking. We used four dichotomous questions to measure participants’ passive smoking avoidance behaviors. The questions included “Would you immediate leave when others smoke near you at home (or in public)?” “Would you ask others stop smoking when they smoke near you at home (or in public)?”

Risk perception is a critical antecedent that affects the decisions of pregnant smokers to quit smoking. If pregnant smokers are not convinced that the risks of smoking threaten the health of their unborn child, they may continue smoking during pregnancy. We investigated the risk perceptions of pregnant smokers and quitters and their attitudes toward smoking during pregnancy. We measured the perceived risk using six items developed by Witte and Morrison [19]. Attitudes toward smoking during pregnancy were measured using three items adapted from the scale developed by Dohnke, Weiss-Gerlach, and Spies [20]. The items of perceived risk and attitudes were measured using a 7-point Likert scale, in which a score of 1 indicated *strongly disagree* and 7 indicated *strongly agree*.

### 2.5. Statistical Analysis Method

We used descriptive statistics to illustrate the demographic data of pregnant smokers and quitters and reveal their smoking and passive smoking behaviors. We compared risk perception, attitude toward smoking during pregnancy, smoking behaviors, and behaviors for avoiding passive smoking between smokers and quitters by using chi-square and *t*-test.

## 3. Results

### 3.1. Sample

The average age of the participants was 30.01 years (SD = 4.92; range 19–44 years), and 84.4% of the participants were in the year range of 21 to 35 (Table 1). Of the participants, 105 (63.3%) were pregnant for the first time. The chi-square and *t*-tests were performed to compare the smoker and quitter groups, which differed in age. The average age of the quitters was 29.59 (SD = 4.84), and the average age of the smokers was 31.33 (SD = 4.97). The smokers were significantly older than quitters (*t* = 2.55, *p* < 0.01; one-tailed). A significant difference existed between the groups in gravidity (*t* = 2.01, *p* < 0.05). The average gravidity of the smokers was 1.70 (SD = 0.99), which was greater than that of the quitters (average = 1.42, SD = 0.66).

### 3.2. Smoking History and Volume

Table 2 lists the smoking histories of the participants. The average smoking history was 6.65 years (SD = 5.10; range 0.5–24 years). Overall, 48.19% participants smoked more than one pack of cigarettes per week prior to their pregnancies. Furthermore, 40 (24.1%) participants continued smoking during pregnancy and 126 (75.9%) quit smoking during pregnancy. Of the quitters, 34 (20.5% of the participants) quit before pregnancy and 92 (55.4% of the participants) quit after becoming pregnant.

**Table 1:** Demographic profile of participants and their smoking behavior.

	Quitters (n = 126)	Pregnant smokers (n = 40)	*t*-test	*p*-value
*Age (years)*				
Average	29.59 years (SD = 4.84)	31.33 years (SD = 4.97)	*t* = −2.55	*p*< 0.01
Below 20	5 (3.9%)	0 (0.0%)		
21–25	29 (23.0%)	3 (7.5%)		
26–30	31 (24.6%)	13 (32.5%)		
31–35	47 (37.3%)	17 (42.5%)		
Above 36	14 (11.1%)	7 (17.5%)		
*Gravidity*				
Average	1.42 (SD = 0.66)	1.70 (SD = 0.99)	*t* = 2.01	*p* = 0.05
Primigravida	83 (65.9%)	22 (55.0%)		
2	32 (25.4%)	12 (30.0%)		
3	10 (7.9%)	3 (7.5%)		
4	1 (0.8%)	2 (5.00%)		
5	0 (0.0%)	1 (2.50%)		

**Table 2:** Comparison between the groups of participants who were quitter and pregnant smoker in terms of smoking history and volume (n = 166).

	Quitters (n = 126)	Pregnant smokers (n = 40)	*t*-test	*p*-value
*Smoking history (years)*				
Average	5.63 years (SD = 4.69)	9.70 years (SD = 5.19)	*t* = 4.66	*p* < 0.01
1 or less	26 (20.6%)	2 (5.0%)		
2–3	30 (23.8%)	5 (12.5%)		
4–5	24 (19.1%)	1 (2.5%)		
6–10	27 (21.4%)	17 (42.5%)		
11–15	15 (11.9%)	12 (30.0%)		
More than 16	4 (3.2%)	3 (7.5%)		
*Smoking volume—prior to pregnancy (pack/week)*				
Average	3.17 pack/week (SD = 3.59)	4.95 pack/week (SD = 4.27)	*t* = −3.00	*p* < 0.01
1	70 (55.6%)	10 (25.0%)		
2	15 (11.9%)	9 (22.5%)		
3	11 (8.7%)	3 (7.5%)		
4 or more	30 (23.8%)	18 (45.0%)		
*Smoking volume—during pregnancy (pack/week)*				
Average	0 pack/week (SD = 0)	3.20 pack/week (SD = 3.70)	*t* = −10.13	*p* < 0.01
*Quit during pregnancy*	*126 (100.0%)*	*0 (0.0%)*		
Less than 1	0 (0.0%)	31 (77.5%)		
1	0 (0.0%)	4 (10.0%)		
2 or 3	0 (0.0%)	1 (2.5%)		
4 or more	0 (0.0%)	4 (10.0%)		

The smokers and quitters differed significantly in smoking history (*t* = 4.66; *p* < 0.01; two-tailed). The quitters smoked for an average of 5.63 years (SD = 4.69), but 63.5% of the quitters smoked for less than 5 years. The smokers smoked for an average of 9.70 years (SD = 5.19), and 80% of the smokers smoked more than 6 years. The results show that the women who quit smoking after pregnancy were typically those with shorter smoking histories compared with those who continued smoking during pregnancy.

The smokers and quitters differed significantly in the smoking volume before pregnancy (*t* = 3.00; *p* < 0.01; two-tailed). The quitters smoked 3.17 packs per week on average (SD = 3.59) before pregnancy. Furthermore, 55.6% of the quitters smoked less than 1 pack per week before pregnancy. The smokers smoked 4.95 packs per week on average (SD = 4.27) before pregnancy; however, the smoking volume of the smokers declined significantly to 3.20 (SD = 3.70) packs per week during pregnancy (*t* = 11.13; *p* < 0.01; one-tailed). Overall, 45% of the smokers smoked more than four packs per week before pregnancy, but 77.5% of the smokers smoked less than one pack per week during pregnancy. Although it is difficult for smokers with long smoking histories and a high smoking volume to quit smoking, pregnant smokers attempt to reduce their smoking volume.

### 3.3. Passive Smoking

Table 3 shows the exposure of the participants to passive smoking. Both the pregnant smokers and quitters in the study typically lived in environments in which they were exposed to passive smoking. Only seven of the women (4.2%) lived with smoke-free families; the remaining 159 participants (95.8%) lived in smoking households. Most of the participants (133, 80.1%) lived with smoking partners. Of the 133 smoked partners, 107 avoided smoking near their own pregnant partner, and 26 ones smoked in the presence of their own pregnant partner. Most of the participants lived with smoking relatives who smoked, and 131 (78.9%) lived with close relatives who were smokers. Of the 131 participants whose relatives were smokers, 34 (25.9%) of the participants had relatives who avoided smoking near them and 97 (74.1%) had relatives who continued smoking in their presence. The results revealed that, even if pregnant women quit smoking, they lived in tobacco-smoke environments because their partners and family members continued smoking.

We also investigated the avoidance behaviors of the participants toward passive smoking. The results revealed that 130 (78.3%) of the participants adopted avoidance behavior toward passive smoking, including immediately leaving smoke-filled environments or asking their family members not to smoke near them. The remaining 36 (21.7%) participants did nothing when their partners or live-in relatives smoked near them. In addition, 138 (83.1%) of the participants avoided smokers in public or asked them not to smoke. The remaining 28 (16.9%) participants did nothing when others smoked near them. The results revealed that certain smokers and quitters did nothing when exposed to households or public environments containing secondhand smoke.

We used a chi-square test to examine the difference between the passive smoking status and anti-passive smoking behaviors of the participants. A significant difference existed between the groups in anti-passive smoking behaviors at home (χ^2^ [df = 1, n = 166] = 20.68, *p* < 0.01) and in public (χ^2^ [df = 1, n = 166] = 20.11, *p* < 0.01).

**Table 3:** Passive smoking of participants.

	Quitters (n = 126)	Pregnant smokers (n = 40)	χ^2^	*p*-value
*Household smoking status*				
Smoke-free family	7 (5.6%)	0 (0.0%)	0.02	0.88
Smoking family	119 (94.4%)	40 (100.0%)		
*Passive smoking—partner*				
Yes	98 (77.8%)	35 (87.5%)	1.61	0.60
No	28 (22.2%)	5 (12.5%)		
*Passive smoking—live together relatives*				
Yes	100 (79.4%)	31 (77.5%)	0.05	0.82
No	26 (20.6%)	9 (22.5%)		
*Action toward passive smoking at home*				
Adopt proactive anti-smoking behavior	109 (86.5%)	21 (52.5%)	20.68	<0.01
—immediately leave	76 (60.3%)	15 (37.5%)		
—ask others stop smoking	33 (26.2%)	6 (15%)		
Do nothing	17 (13.5%)	19 (47.5%)		
*Action toward passive smoking in public*				
Adopt proactive anti-smoking behavior	114 (90.5%)	24 (60.0%)	20.11	<0.01
—immediately leave	110 (87.3)	24 (60.0%)		
—ask others stop smoking	4 (3.2%)	0 (0.0%)		
Do nothing	12 (9.5%)	16 (40.0%)		

### 3.4. Scale Reliability and Validity

We used multi-item scales to measure the attitudes and risk perceptions of the participants and calculated the Cronbach alphas and composite reliability to measure the reliability of the scales. Table 4 indicates that the Cronbach alpha and composite reliability values exceed 0.70, which is within the acceptable range. We assessed the convergent validity by examining the average variance extracted (AVE) of each dimension. The results showed that the AVE values in the current study were above the value of 0.5 suggested by Fornell and Larcker [21]. Therefore, we confirmed the convergent validity of the measurement scales.

### 3.5. Risk Perceptions and Attitudes toward Smoking

We used *t*-test analysis to investigate whether significant differences existed in risk perception and attitude toward smoking during pregnancy between groups of quitters and smokers. Table 5 shows that significant differences were discovered in risk perception. The quitters had a greater awareness of the threat of smoking to fetal health (x_quitter_ = 6.46 > x_smoker_ = 6.09, *p* < 0.05; one-tailed), severity of the threat of smoking to fetal health (x_quitter_ = 6.35 > x_smoker_ = 5.72, *p* < 0.01; one-tailed), and overall perceived risk (x_quitter_ = 6.40 > x_smoker_ = 5.90, *p* < 0.01; one-tailed) compared with the smoker group.

The results revealed that perceived risk is a critical antecedent affecting the decisions of women to quit smoking. Pregnant women quit smoking when they perceived that smoking causes a high level of risk to fetal health. The results revealed no significant difference between smokers and quitters in their attitudes toward smoking during pregnancy.

**Table 4:** Scale reliabilities and validity.

Variables	No of Items	Factor loading	Cronbach α	Composite Reliability	Average Variance Extracted (AVE)
Attitude	3	0.796	0.810	0.848	0.655
		0.856			
		0.929			
Perceived risk —overall risk perception	6	0.794	0.945	0.941	0.723
		0.898			
		0.864			
		0.939			
		0.932			
		0.928			
Perceived risk —susceptibility to threat	3	0.897	0.899	0.916	0.784
		0.958			
		0.899			
Perceived risk —severity of the threat	3	0.983	0.983	0.982	0.947
		0.984			
		0.983			

**Table 5:** Risk perception and attitude to anti pregnancy smoking.

	quitters (n = 126)	smokers (n = 40)	*t*-value	*p*-value
Attitude	6.58 (SD = 0.87)	6.33 (SD = 0.98)	1.47	0.15
Perceived risk—overall risk perception	6.40 (SD = 0.85)	5.90 (SD = 1.01)	3.08	<0.01
Perceived risk—susceptibility to threat	6.46 (SD = 0.77)	6.09 (SD = 0.98)	2.43	0.01
Perceived risk—severity of the threat	6.35 (SD = 1.04)	5.72 (SD = 1.21)	3.22	<0.01

We used *t*-test analysis to investigate the differences in risk perceptions and attitudes toward smoking during pregnancy between women who adopted and did not adopt anti-passive smoking behaviors; Table 6 shows the significant differences in attitudes and risk perceptions between women who adopted and did not adopt anti-passive smoking behaviors. Compared with women who took no anti-passive smoking actions, women who adopted anti-passive actions at home had more positive attitudes toward avoiding smoking during pregnancy (x_Avoid passive smoking_= 6.62 > x_do nothing_ = 6.16, *p* < 0.05; one-tailed), higher levels of risk perception of threat susceptibility (x_Avoid passive smoking_ = 6.49 > x_do nothing_ = 5.92, *p* < 0.01; one-tailed), higher levels of risk perception of the severity of the threat (x_Avoid passive smoking_ = 6.32 > x_do nothing_ = 5.77, *p* < 0.05; one-tailed), and higher levels of overall perceived risk (x_Avoid passive smoking_ = 6.40 > x_do nothing_ = 5.84, *p* < 0.01; one-tailed). The results reveal that pregnant women with high levels of anti-smoking attitudes and high risk perceptions of smoking during pregnancy adopt avoidance behaviors to passive smoking at home.

**Table 6:** Risk perception and avoidance behaviors towards anti passive smoking.

	Anti-passive smoking action in household			Anti-passive smoking action in public places		
	Adopt proactive action (n = 130)	Do nothing (n = 36)	*t*-value (*p*-value)	Adopt proactive action (n = 138)	Do nothing(n = 28)	*t*-value (*p*-value)
Attitude	6.62 (SD = 0.83)	6.16 (SD = 1.05)	2.76 (*p* = 0.01)	6.63 (SD = 0.81)	5.95 (SD = 1.11)	3.78 (*p* < 0.01)
Perceived risk—overall risk perception	6.40 (SD = 0.84)	5.84 (SD = 1.04)	3.37 (*p* < 0.01)	6.41 (SD = 0.83)	5.65 (SD = 1.05)	3.60 (*p* < 0.01)
Perceived risk—susceptibility to threat	6.49 (SD = 0.76)	5.92 (SD = 0.94)	3.37 (*p* < 0.01)	6.47 (SD = 0.77)	5.86 (SD = 0.96)	3.19 (*p* < 0.01)
Perceived risk—severity of the threat	6.32 (SD = 1.04)	5.77 (SD = 1.26)	2.66 (*p* = 0.01)	6.35 (SD = 1.02)	5.45 SD = 1.26)	4.06 (*p* < 0.01)

We found similar results for anti-passive smoking in public. Women who adopted anti-passive smoking actions in public had more positive attitudes toward avoiding smoking during pregnancy (x_Avoid passive smoking_ = 6.63 > x_do nothing_ = 5.95, *p* < 0.01; one-tailed), higher levels of risk perception of threat susceptibility (x_Avoid passive smoking_ = 6.47 > x_do nothing_ = 5.86, *p* < 0.01; one-tailed), higher levels of risk perception of the severity of the threat (x_Avoid passive smoking_ = 6.35 > x_do nothing_ = 5.45, *p* < 0.01; one-tailed), and higher levels of overall perceived risk (x_Avoid passive smoking_ = 6.41 > x_do nothing_ =5.65, *p* < 0.01; one-tailed) compared with those who did nothing to avoid passive smoking.

Based on results of this empirical survey, attitudes toward smoking during pregnancy and risk perceptions were antecedents to passive smoking avoidance behaviors. At home and in public, pregnant women with more positive attitudes toward avoiding smoking during pregnancy and higher levels of risk perception adopted more passive smoking avoidance behaviors than did those with more negative attitudes toward avoiding smoking during pregnancy and low levels of risk perception.

## 4. Discussion

In the current study, we used a questionnaire to explore the perceptions, attitudes, and avoidance behaviors toward the smoking and passive smoking of pregnant Taiwanese smokers and recent quitters. The study revealed that approximately three-fourths of pregnant women quit smoking during their pregnancy, but the remaining one-fourth continued smoking. In accordance with previous research, the study revealed that pregnant smokers were susceptible to the smoking behaviors of their family members. In this study, all the pregnant smokers lived in smoking households with partners or relatives who smoked. Researchers have suggested that women believe that removing themselves from the vicinity of smokers effectively reduces their exposures to passive smoking [22]. However, this is not entirely correct. Only smoke-free environments sufficiently prevent the hazards of exposures to passive smoking.

The fetuses of these women had increased risks of health problems caused by tobacco smoke pollution. Numerous pregnant women who quit smoking were exposed to passive smoking. More than 94% of recent quitters lived in smoking households with partners or relatives who smoked, and more than 74% lived with relatives who continued smoking near them. Thus, pregnant women who recently quit smoking continued to be exposed to environmental smoke. Fetal health did not appear to motivate family members to quit smoking. Nevertheless, fetal health is a critical reason for pregnant women to quit smoking, and most of the quitters chose to quit smoking after becoming pregnant.

It is likely difficult for pregnant women with long smoking histories to quit smoking. Compared with recent quitters, pregnant smokers had a longer history of smoking. In the present study, the pregnant smokers had smoked for 6–10 years whereas the quitters smoked for less than six years. The group of smokers had a longer history of smoking compared with the quitters. This supports the argument that quitting is more difficult for a person who smokes for a long time than one person who smokes for a short time. Furthermore, Dohnke, Weiss-Gerlach, and Spies suggested that the volume of smoke intake is correlated with the intention to quit smoking [20]. Consistent with previous studies, we found that the smokers smoked more than four packs of cigarettes per week before their pregnancy. By contrast, most quitters smoked less than one pack per week before pregnancy. Although the smokers could not completely quit smoking during their pregnancies, their smoking volume significantly decreased.

The current study also revealed that most quitters adopted proactive anti-smoking behaviors to confront passive smoking at home and in public. These avoidance behaviors included immediately leaving smoke-filled areas or requesting others stop smoking. Although the smokers did not quit smoking, some adopted anti-passive smoking behaviors by asking others not to smoke near them or removing themselves from areas where others were smoking. The results revealed that many pregnant women were aware of the health risks of secondhand smoke to their unborn babies. Pregnant women adopted behaviors such as reducing their smoking volume and adopting passive smoking avoidance behaviors to protect their unborn babies.

The results of the empirical survey indicate that women who continued smoking during pregnancy perceived fewer health risks associated with smoking compared with those who quit. Thus, perceived risk predicts the motivation of pregnant women to avoid tobacco use. Women who quit smoking during pregnancy may realize that continued smoking can harm their unborn baby.

The perception that smoke damages the health of pregnant women and their unborn babies is a critical antecedent to avoid both active and passive smoking. Compared with those who did nothing to avoid passive smoking, we determined that women who adopted passive smoking avoidance behaviors had a greater perception of the risk of smoking on fetal health, and held more positive attitudes toward anti-passive smoking both at homes and in public spaces. Furthermore, pregnant women who did nothing to avoid passive smoking perceived that the fetal health risk from tobacco pollution was lower than did those who adopted passive smoking avoidance behaviors. Researchers have reported that partners who are non-smoking benefit pregnant women’s attempts to avoid passive smoke [23]. In accordance with previous literature, women who smoked before their pregnancies typically came from smoking families, had partners who smoked, or lived with relatives who smoked. It is essential that pregnant women and their partners and close relatives be educated on the health risks of active and passive smoking to both their babies and the women themselves.

### 4.1. Contributions and Implications

We investigated the perceptions of pregnant women regarding the risks of smoking during pregnancy. Researchers have often discussed the influence of demographics, knowledge, and self-efficacy on the behaviors of women in quitting smoking and avoiding the harm of passive smoking. Scant research has considered the perceptions of pregnant smokers regarding the health risks of smoking and the avoidance of passive smoking. The current study revealed that pregnant quitters may continue to be exposed to environmental tobacco smoke after quitting. The results also indicated that perceived fetal health risks are critical antecedents to both quitting active smoking and avoiding passive smoking. The probability that pregnant smokers quit smoking and adopt passive smoking avoidance behaviors is positively correlated with the perceived fetal health risks of smoking. If pregnant smokers are not aware of the health risk of smoking, they may continue to smoke and expose themselves to passive tobacco pollution.

We also investigated the avoidance behaviors toward passive smoking of pregnant Taiwanese smokers and quitters. The results demonstrated that most of the smokers and quitters lived with families who smoked, but attempted to avoid and did not want to be victims of passive smoking. The results demonstrate the importance of promoting smoking cessation at home and in public to protect fetuses and babies from environmental tobacco smoke pollution.

The study extended our knowledge of the active smoking and avoidance behaviors to the passive smoking of pregnant smokers and quitters. The results are relevant to government agencies or anti-smoking organizations that advocate tobacco control. Those who champion avoiding smoking during pregnancy should use advertising and publicity to advise pregnant women of risks, enhancing their understanding of the harm of tobacco and secondhand smoke. This increased risk perception can help pregnant women avoid smoking and environmental tobacco smoke pollution.

### 4.2. Limitations

The conclusions drawn in this study are based on a convenience sample of pregnant women in an online baby-parenting community; thus, the generalizability of the results is limited. However, we collected data in the most well-known online baby-parenting community in Taiwan. This could rule out the possibility of recruiting the non-target group as participants in this study; thus increasing the representativeness of the sample. Another limitation in this study resulted from using self-reported questionnaires. The qualitative information generated using self-reported questionnaires is typically reliable, but the responses are affected by recall bias.

## 5. Conclusions

Smoking during pregnancy is significantly associated with increased health risks. To reduce smoking during pregnancy, we should realize the factors influencing the attitudes of pregnant smokers toward smoking and passive smoking. This study investigated the perceptions of pregnant smokers and quitters regarding the risks of smoking during pregnancy. We concluded that most women stop smoking during pregnancy; however, most of them continue to be exposed to passive-smoking environments. It is essential that pregnant women and their partners and close relatives be educated on the health risks of active and passive smoking to both their babies and the pregnant women themselves. Only smoke-free environments sufficiently prevent the hazards of exposures to passive smoking.
